# Waiting Times and Influencing Factors in Children and Adults Undergoing Assessment for Autism, ADHD, and Other Neurodevelopmental Differences

**DOI:** 10.1002/aur.70011

**Published:** 2025-02-24

**Authors:** Donald Maciver, Anusua Singh Roy, Lorna Johnston, Marie Boilson, Eleanor Curnow, Victoria Johnstone‐Cooke, Marion Rutherford

**Affiliations:** ^1^ National Autism Implementation Team, School of Health Sciences Queen Margaret University Musselburgh UK; ^2^ Division of Occupational Therapy and Arts Therapies, School of Health Sciences Queen Margaret University Musselburgh UK; ^3^ Dublin South Central Mental Health Services, CHO 7 National Clinical Programme for Adult ADHD for Ireland, Health Service Executive Dublin Republic of Ireland

**Keywords:** assessment, autism, diagnosis, neurodevelopmental, pathway

## Abstract

This study explored waiting times and the factors influencing them in child and adult populations undergoing assessment for autism, ADHD, and other neurodevelopmental differences. The analysis focused on a retrospective review of 408 cases with assessments completed between October 2021 and May 2022, conducted by 30 diagnosing teams in Scotland. Data included age, final diagnosis, demographics, medical and developmental history, contact frequency, and assessment service adherence to best‐practice standards. Waiting times were calculated, and relationships were analyzed using linear regression. Median waiting times were 525 days (IQR 329–857) for children/adolescents and 252 days (IQR 106–611) for adults. Only 20% of children's and 47% of adult assessments met the proposed 252‐day diagnostic time target. Autism and ADHD were the most common diagnoses. Receiving > 1 neurodevelopmental diagnosis on completion was uncommon. Demographic factors did not significantly affect waiting times. Children/adolescents with more complex developmental and medical histories experienced longer waits (100.3 weeks vs. 67.7 weeks; *p* < 0.001), while adults with similar histories had shorter waits (32.7 weeks vs. 57.4 weeks; *p* = 0.016). Adults with ADHD experienced longer waits than autistic adults (63.4 weeks vs. 38.6 weeks, *p* = 0.002). Adherence to best‐practice quality standards was associated with shorter waits for children (β = 0.27, p = 0.002), but the relationship between standard adherence at different stages and for adults was less clear. More frequent appointments correlated with shorter adult waits (33.7 weeks vs. 59.2 weeks, *p* = 0.015). Gender distribution was balanced among adults, but children's services included more boys. The study highlights long waits and the need for improvement in processes.

**Summary**

- This study looked at how long neurodivergent people have to wait for assessments and diagnoses.
- The researchers examined medical records of 408 people (206 children and 202 adults) in Scotland.
- They found that waiting times were quite long, with children and adolescents waiting an average of 525 days and adults waiting 252 days to get a diagnosis.
- Only about 20% of children and less than half of adults received their diagnoses within the recommended 252 days.
- The study did not find that ethnicity, gender, or language affected waiting times.
- However, children with more complex health and developmental issues had to wait longer, while adults with similar issues waited less.
- Adults who recieved a diagnosis of ADHD generally waited longer than those who recieved a diagnosis of autism.
- The study also looked at how well the teams adhering to quality standards could affect waiting times.
- For children, better adherence to these standards was associated with shorter waiting times, but for adults, the picture was more mixed.
- Having more frequent appointments was linked with shorter waits for adults.
- The research highlights a need to make the process of getting a neurodevelopmental assessment faster.
- The authors suggest that changing the way assessment is carried out, for example, by having a joint pathway for autism and ADHD, could help reduce wait times and improve the quality of services.

Despite guidelines aimed at expediting neurodevelopmental diagnosis, the process is often sluggish and taxing (Arnold et al. 2020; Bonati et al. 2019; Boshoff et al. 2019; McGill et al. 2020; Young et al. 2021). Timely diagnosis is not a convenience; it significantly impacts lives. Feedback from neurodivergent individuals highlights the importance of diagnosis for understanding, access to support, connecting with others, and clarifying why previous supports were ineffective (Arnold et al. 2020; de Broize et al. 2022; McGill et al. 2020; Zener 2019). Historically, diagnosis has focused on autistic male children. This contrasts with recent increases in adults, particularly women, seeking assessment (Russell et al. 2022; Young et al. 2020). In tandem, moving away from isolated diagnosis toward a comprehensive cross‐neurotype assessment is advocated (Abrahamson et al. 2021; Curnow, Utley, Rutherford, Johnston, and Maciver, 2023; Lang et al. 2024; Maciver et al. 2022; Rutherford and Johnston 2023; Rutherford et al. 2021). There are distinct differences, for example, between autism and ADHD (Antshel and Russo 2019), but transdiagnostic multidisciplinary approaches are important, as many neurodivergent individuals will have traits that cross several labels (Gillberg 2021). The “ESSENCE” framework provides a foundation (Gillberg 2021), and the DSM‐5 (American Psychiatric 2013) and ICD‐11 (World Health Organisation 2018) now allow for co‐occurring neurodevelopmental diagnoses.

There remain significant international variations in the time taken to receive a diagnosis (Rocco et al. 2021). It is often difficult to determine factors that contribute to delays. While early and reliable diagnosis of neurodevelopmental differences is possible in early childhood and infancy (Dawson et al. 2023), differences can remain undiagnosed until school age and adulthood (van’t Hof, Tisseur, and van Berckelear‐Onnes, 2021). The diagnostic process is complicated by funding, a shortage of professionals, and inconsistency (Male, Farr, Bremner, et al. 2023). Adult diagnostic services are more limited than those for children, with challenges including a gap between capacity and demand and the absence of best‐practice guidance (Young et al. 2021). In Scotland and the United Kingdom, access to adult neurodevelopmental assessment typically requires the presence of a moderate to severe mental illness, as assessment is usually through specialist mental health services (Katzman et al. 2017). This restricts adults from accessing diagnosis unless they are in crisis.

Factors driving waits are complex to evaluate. Some studies rely on administrative data, lacking the clinical depth necessary. Others focus on clinical factors, neglecting aspects related to service provision and quality. Integrating clinical history details with service provision factors is needed. A key direction is to move beyond the small populations typically available within trials or research cohorts and focus instead on real‐world clinical data. Historically, most research has focused on children (Asherson et al. 2022), leaving gaps in understanding for adults.

## 1 Aims of the Current Study

The Scottish Government funded the National Autism Implementation Team (NAIT), authors of this study, to develop pathways that improve outcomes for neurodivergent people. The team includes neurodivergent and neurotypical members, along with professionals from Psychiatry, Education, Speech and Language Therapy, and Occupational Therapy. They collaborate with academics and service users to promote evidence‐informed, neuro‐affirming practices through multi‐professional and cross‐sector partnerships. The team has published research and best‐practice guidance, including standards for assessment and diagnosis timing (Curnow, Utley, Rutherford, Johnston, and Maciver, 2023; Curnow, Rutherford, Maciver, et al. 2023; Maciver et al. 2022, 2023; National Autism Implementation Team 2021, 2023; Rutherford 2020; Rutherford and Johnston 2023; Rutherford et al. 2021).

The aims of this study were threefold: to evaluate waiting times in children and adults, to analyze the impact of demographic and clinical factors on waiting times, and to explore how service quality process factors and adherence to best‐practice guidelines influence waiting times. This exploratory investigation hypothesized that waiting times would be influenced by a combination of demographic, clinical history, and service‐level factors.

## 2 Materials and Methods

### 2.1 Setting

Scotland is the second largest country in the United Kingdom and has a population of over 5 million. Scotland has a robust publicly funded education system for children aged 3–18. Its state health system provides comprehensive services, and private alternatives, especially for children, are limited. However, for children and adults, existing services are in high demand, and in many places, this is not matched by capacity. Educational policy promotes inclusion, aiming for universal mainstream education, but national consistency remains a challenge due to substantial local differences in practices. For children with neurodevelopmental differences, assessment and support are facilitated through education or health monitoring, or self‐referral, with diagnosis provided by multidisciplinary teams including doctors and allied health professionals (including nurses, occupational therapists, psychologists, speech and language therapists, and social work or social care). Adults seeking diagnosis rely on general practitioners (family doctors) as gatekeepers to secondary care specialists in mental health, with diagnosis provided by individual psychiatrists or by multidisciplinary teams (including mental health practitioners, occupational therapists, nurses, and psychologists). For adults, long‐term management typically occurs in primary care, sometimes in collaboration with psychiatry, mental health services, and allied health professionals. In some areas, support is available through commissioned third‐sector services (charities). For adults, the lack of services is compounded by a lack of skills and knowledge in staff.

### 2.2 Study Design

The study employed a cross‐sectional retrospective case note review to investigate diagnostic practices across Scotland's 14 health boards (geographical entities responsible for health administration) from adult and child/adolescent diagnosing teams.

### 2.3 Sample

All health boards were approached, with voluntary participation sought. Participation was confirmed by senior managers, with 10/14 boards consenting (4 boards declining and citing work pressures). In the absence of a central register of diagnosing teams, managers within boards identified and volunteered teams that routinely conduct neurodevelopmental assessments. Once teams had been identified, team‐specific case quotas were established based on their size and likely referral rate. Diagnosing teams were classified as either “adult” or “child/adolescent” services based on their service classification, and cases were labeled as either child/adolescent or adult according to the team handling them. Most individuals in the “child” services category were aged 0–18 years at referral, while those in the “adult” services category were typically over 18 years old at referral. However, some individuals in the “adult” services category were under 18 at referral, as individuals may transition to “adult” services earlier than 18.

The inclusion criteria for cases were as follows. For young people's services: individuals referred for autism, fetal alcohol spectrum disorder, attention deficit hyperactivity disorder (ADHD), developmental language disorder, developmental coordination disorder, and/or intellectual disability assessment. For adult services: individuals referred for assessment for autism, ADHD and/or intellectual disability assessment.

Each diagnosing team compiled all cases meeting the inclusion criteria completed within the preceding 12 months. To ensure the data captured contemporary practices and outcomes, cases were selected for inclusion consecutively in reverse chronological order, commencing with the most recently completed case and proceeding backwards until the required quota was met. Teams participated between October 2021 and May 2022.

### 2.4 Data

Data extraction was via a digital form. This form was validated by a panel of experts specializing in neurodevelopmental assessment and underwent a pilot phase with clinicians for accuracy and user‐friendliness. The form collected a comprehensive dataset, including demographics, timestamps of events, clinical history, and metrics on the diagnostic process (e.g., number and type of clinical interactions, assessment tools, and team specification). Local staff performed data extraction from on‐site clinical notes.

### 2.5 Measures

#### 2.5.1 Main Outcome: Waiting Times

Waiting times were calculated as follows: (1) wait for first appointment (wait from referral to the first meeting with the diagnosing team); (2) assessment duration (wait from the first meeting to the last meeting with the diagnosing team); wait for diagnosis to be communicated (wait from the end of the final meeting to written confirmation of diagnosis); and (3) total waiting time (the full period from initial referral to final diagnosis being formally shared). The total waiting time was used in all analyses as the outcome variable; other waiting times are presented descriptively.

### 2.6 Other Variables Used in Modeling

#### 2.6.1 Final Diagnosis Received

Variables used to categorize cases were (1) autism with or without another diagnosis (excluding ADHD); (2) ADHD with or without another diagnosis (excluding autism); (3) autism and ADHD combined, with or without another diagnosis; and (4) other (any other outcome including no diagnosis). Analysis focused on autism and ADHD, as the most common diagnoses.

#### 2.6.2 History Factors

Medical and developmental history factors previously found to influence neurodevelopmental diagnosis were included: neurological disorder (Socanski et al. 2013); intellectual disability (Huang et al. 2020); speech delay (Nitzan et al. 2023) or regression (Boulton et al. 2023); being born preterm (classified as 35 weeks or below) (Chung et al. 2020); additional support in school (Sapiets et al. 2021); genetic family history of neurodevelopmental diagnosis (Oliva et al. 2021); genetic family history of a neurological condition (Xie et al. 2019); and (for adults only) involvement in supported social care or supported housing (Burke et al. 2019). To manage the volume of variables in the analysis, these were combined to provide a score of 0–8 for adults and 0–7 for children, with a score of 7 or 8 indicating the presence of all factors and a score of 0 indicating that none were present.

#### 2.6.3 Demographic Factors

We included three demographic factors that may present risk for individuals in receiving a timely diagnosis. These were female sex (Knott et al. 2024; McDonnell et al. 2021; Wallisch et al. 2021), minority ethnic group (Dababnah et al. 2018; Overs et al. 2017) (defined, using the terminology used by the Scottish Government and NHS, as any mixed or multiple minority ethnicity, Pakistani, Pakistani Scottish or Pakistani British, Chinese, Chinese Scottish or Chinese British, African, African Scottish or African British, and Other non‐white ethnicity), and primary language spoken at home not being English (Collins et al. 2017). We created a composite “risk” variable (0–3) to account for intersectional effects, where 3 indicated the presence of all three demographic factors and 0 indicated none. Given the smaller overall sample size, particularly among ethnic minority participants, we could not analyze these factors independently. Additionally, as most individuals in this category were female, we conducted separate analyses using female sex as a standalone variable. The results were consistent with the combined variable.

#### 2.6.4 Number of Contacts

Defined as the sum of the number of direct and indirect contacts with the diagnosing team following referral, including face‐to‐face, phone call, or video conference.

#### 2.6.5 Adherence to Process Quality Standards

Standards were derived from a national practice framework developed for pediatric neurodevelopmental diagnosis (Rutherford et al. 2021) (Table 1). Due to the absence of standards for adults, children's standards were applied universally. The framework offered recommendations addressing the quality and timing of the assessment and diagnosis covering the pre‐referral stage and assessment. Under the guidance of the clinician who led the framework development (MR), key aspects were identified and condensed into 13 concise statements (4 standards for the pre‐referral phase, 9 standards for the assessment phase) (see Table 1 and Additional File 1 for details). These were used by the researchers to assess adherence for each case, coding each standard as “Yes” (met) or “No” (not met or unclear). The level of adherence was quantified using “Yes” scores. The standards were utilized in three ways in the analysis: (1) full standards covering the entire diagnostic process with possible scores ranging from 0 to 13, (2) pre‐referral standards alone with possible scores ranging from 0 to 4, and (3) assessment standards alone with possible scores ranging from 0 to 9. Adherence to time standards was analyzed, with guidelines set by Rutherford et al. (2021) for Scotland. Due to multi‐collinearity and violation of the independence assumption, adherence to these standards was excluded from the multivariable regression and presented descriptively.

**TABLE 1:** Quality standards (derived from Rutherford et al. (2021)).

Pre‐referral standards	The pre‐referral process included a developmental and family history
	2 The pre‐referral process included information drawn from a variety of contexts, such as home, school, workplace, and the community
	3 The pre‐referral process included indicators for concern identified through a screening tool
	4 The pre‐referral process included information drawn from direct observation or interview with the individual
Assessment standards	The neurodevelopmental assessment was conducted by more than one person as part of an MDT
	2 Diagnosis was made with reference to recognized diagnostic criteria (DSM or ICD)
	3 At the time of diagnosis, post‐diagnosis information was given
	4 A post‐diagnosis follow‐up meeting was offered
	5 The neurodevelopmental diagnostic assessment included history taking
	6 The neurodevelopmental diagnostic assessment used information drawn from observation of the individual
	7 The neurodevelopmental diagnostic assessment included a parent/carer/partner/family member
	8 The neurodevelopmental diagnostic assessment used information drawn from a variety of contexts, such as home, school, workplace, and community
	9 The neurodevelopmental diagnostic assessment used information drawn from standardized or other formal tool
Time standards	Request for neurodevelopmental assessment (pre‐referral): time from acceptance of referral to the first appointment should be no longer than 12 weeks
	2 Diagnostic assessment: Time from the first appointment to the last appointment should be no longer than 22 weeks
	3 Full process total waiting time (from request/referral accepted to diagnosis shared) should be no longer than 36 weeks

Abbreviations: DSM, Diagnostic and Statistical Manual of Mental Disorders; ICD, International Classification of Diseases.; MDT, Multidisciplinary Team.

#### 2.6.6 Statistics

Analyses were conducted in R (R Foundation, Vienna, Austria). Missing data were present in fewer than 10% of observations. We conducted a complete case analysis using listwise deletion where required. Modeling was conducted for children/adolescents' services and adult services separately. Ordinary least‐squares (OLS) multiple linear regression models (Montgomery et al. 2021) were employed to examine the relationship between waiting time (dependent variable) and the independent variables.

Variables were defined as previously described. Additional groups were derived as follows. The distributions were examined, and cut‐offs were established to ensure balanced groups, where possible. Referral and assessment quality standard adherence were classified into three categories based on the proportion of standards met: for referral—“Lower” (0–1 standards met), “Moderate” (2 standards met), or “Higher” (3–4 standards met). Assessment adherence cut‐offs were defined as follows: for adults—“Lower” (0–4 standards met), “Moderate” (5–7 standards met), or “Higher” (8–9 standards met); for children—“Lower” (0–5 standards met), “Moderate” (6–7 standards met), or “Higher” (8–9 standards met). History factors were classified based on the proportion of factors present, categorized as “Lower” (1–3 factors present), “Higher” (4 or more factors present), or “None” (no factors present) (further details in Tables 5 and 6 and accompanying Supporting Information). Examination of distributions showed that the dependent variable waiting time (in weeks) was positively skewed. Therefore, a logarithmic transformation (Benoit 2011) was performed.

The independent variables of final diagnosis, demographic factors, history factors, and number of contacts were included in all models. For adherence to process quality standards, two models were built. Model A consolidated all standards into a single score, while Model B disaggregated standards into pre‐referral and assessment categories to assess stage‐specific impacts. Coefficients using Wald's t‐test and p‐values for each independent variable were obtained. Significance was set at the 5% level. The regression coefficient estimates represented the amount of change in waiting time for a change in the independent variable. Model fit was determined through goodness‐of‐fit statistics and robustness ensured by verifying the linear regression assumptions of independence, linearity, normality, and homoscedasticity (Poole and O'Farrell 1971). *F*‐tests and associated *p* values were used to evaluate the overall fit of the models. Multiple and adjusted *R*‐squared values determined the explanatory power of the models by showing the proportion of variation in the dependent variable that the models can explain. Model assumptions were tested using diagnostic plots of probability density, residuals and standardized residuals and variance inflation factor, and quantile–quantile plots of normality.

#### 2.6.7 Ethical Approval

Ethical approval was received from Queen Margaret University Ethics Committee. Analysis was based on an anonymized dataset containing routinely collected clinical data, so no consents were sought from individuals. Consent for data access was granted by the NHS Scotland Public Benefit and Privacy Panel for Health and Social Care.

## 3 Results

The study covers 10 out of 14 (71%) of Scotland's health boards, representing approximately 4.8 million people, or 89% of the Scottish population. Autism and ADHD were the most common diagnoses. The data collected included 30 diagnosing teams (12 adult and 18 children's teams), 206 children/adolescents (76 female; *M*_age_ at referral = 8.99 years, SD = 4.6, range = 1.4–17.9), and 202 adults (98 female; *M*_age_ at referral = 29.9 years, SD = 10.5, range = 15.8–71.1). There was a high rate of receiving a neurodevelopmental diagnosis (83.01% children; 87.62% adults), but receiving more than one diagnosis was uncommon (21.36% children; 14.85% adults received > 1 diagnosis). Total contacts were 939 for children (*M* = 4.6 contacts; SD = 1.103; range = 1–10) and 724 for adults (*M* = 3.6 contacts; SD = 1.732; range = 1–10). See Table 2 and Additional File 3 for further details.

**TABLE 2:** Demographic characteristics of participants.

Characteristics	Adults (*N* = 202)	Children and adolescents (*N* = 206)
	Mean (SD); Range	Mean (SD); Range
Age at referral (years)	29.9 (10.5); 15.7–71.1	8.99 (4.6); 1.4–17.9
Age at diagnosis (years)	31.2 (10.3); 17.2–71.8	10.6 (4.5); 2.6–19.1

	*n* (%)	*n* (%)
Demographics: Sex		
Female	98 (48.51)	76 (36.89)
Male	99 (49.01)	129 (62.62)
Other/not known	5 (2.48)	1 (0.49)
Demographics: Ethnicity		
White		
– White Scottish	145 (71.78)	130 (61.11)
– White Other British	13 (6.44)	14 (6.80)
– White Polish	2 (0.99)	3 (1.46)
– White Other White	6 (2.97)	4 (1.94)
Minority ethnicity		3 (1.46)
– Any mixed or multiple	2 (0.99)	
– Pakistani, Pakistani Scottish, or Pakistani British	1 (0.50)	0 (0.00)
– Chinese, Chinese Scottish, or Chinese British	2 (0.99)	3 (1.46)
– African, African Scottish, or African British	1 (0.50)	0 (0.00)
– Other ethnicity	1 (0.50)	3 (1.46)
Missing	29 (14.36)	46 (22.33)
Demographics: Primary language at home		
English	192 (95.05)	194 (94.17)
Other language	10 (4.95)	12 (5.83)
Final diagnosis received^a^		
ND diagnosis (any)	177 (87.62)	171 (83.01)
– Autism diagnosis	79 (39.11)	144 (69.90)
– ADHD diagnosis	91 (45.05)	43 (20.87)
– DCD diagnosis	0 (0.00)	5 (2.43)
– ID diagnosis	11 (5.45)	17 (8.25)
– FASD diagnosis	1 (0.50)	0 (0.00)
– DLD diagnosis	2 (0.99)	3 (1.46)
Other non‐ND diagnosis	27 (13.37)	17 (8.25)
No diagnosis Multiple (> 1) diagnoses received	28 (13.86) 30 (14.85)	28 (13.59) 44 (21.36)
History factors		
Neurological disorder	10 (4.95)	4 (1.94)
Intellectual disability	22 (10.89)	22 (10.68)
Speech delay or speech regression	35 (17.33)	90 (43.49)
Born preterm	2 (0.99)	10 (4.85)
Having ASN (educational)	39 (19.31)	119 (57.77)
Social care/housing (adult only)	14 (6.93)	—
Genetic family history (neurodevelopmental)	67 (33.17)	101 (49.03)
Genetic family history (other)	20 (9.90)	40 (19.42)
Total assessment contacts (*n*)		
1	13 (6.44)	7 (3.40)
2	28 (13.86)	50 (24.27)
3	36 (17.82)	64 (31.07)
4	29 (14.36)	28 (13.59)
5	34 (16.83)	18 (8.74)
6	30 (14.85)	28 (13.59)
7	14 (6.93)	4 (1.94)
8	9 (4.46)	1 (0.49)
9	4 (1.98)	1 (0.49)
10 or more	9 (4.46)	1 (0.49)

^a^ Counts of labels; this includes individuals who received multiple diagnoses, so will add to > 100% of cases.

Abbreviations: ADHD, Attention Deficit Hyperactivity Disorder; ASN, Additional Support Needs (educational); CD, Developmental Coordination Disorder; DLD, Developmental Language Disorder; FASD, Fetal Alcohol Spectrum Disorder; ID, Intellectual Disability; ND, Neurodevelopmental.

### 3.1 Waiting Times

There were waits across all stages. Results are in Tables 3 and 4, including the proportion that met the proposed time standards.

**TABLE 3:** Waiting times and adherence to time standards for adults (*n* = 202).

	T1	T2	T3	Full waiting time
Proposed maximum time standard	84 days	154 days	As soon as possible	252 days
Cases meeting standard (%)	45.05	73.27	N/A	47.03
Range (days)	0–1915	0–964	0–233	0–1985
Median (days)	106	56	0	252
IQR (days)	33–346	26–155	0–11	106–611
Mean (days)	244	114	14	365
SD	293.2	148.2	19.1	324.5

*Note*: T1 = wait for the first appointment following referral; T2 = duration of assessment; T3 = wait for diagnosis to be communicated; Full waiting time = T1 + T2 + T3; A score of “0” indicates the identified start date and identified end date for that phase was on the same day.

Abbreviations: IQR, interquartile range; SD, standard deviation.

**TABLE 4:** Waiting times and adherence to time standards for children and adolescents (*n* = 206).

	T1	T2	T3	Full waiting time
Time standard	84 days	154 days	As soon as possible	252 days
Cases meeting standard (%)	43.20	39.32	N/A	19.90
Range (days)	0–1056	0–1393	0–28	33–1462
Median (days)	106	214	0	525
IQR (days)	28–379	63–533	0–27	329–857
Mean (days)	223	337	22	574
SD	254.8	339.8	40.5	332.7

*Note*: T1 = wait for the first appointment following referral; T2 = duration of assessment; T3 = wait for diagnosis to be communicated; Full waiting time = T1 + T2 + T3; A score of “0” indicates the identified start date and identified end date for that phase was on the same day.

Abbreviations: IQR, interquartile range; SD, standard deviation.

### 3.2 Modeling

Results of the analysis of factors associated with waiting times are in Tables 5 and 6. Models were built for children's and adult services and each included two models (model A and model B). Model A included all quality standards as independent variables, and model B included pre‐referral quality standards and assessment quality standards as separated independent variables. All models showed significant improvement over an intercept‐only model (all *F*‐tests for nested models showed a significant *p* < 0.005; ref.: Tables 5 and 6) and were a better fit to the data, implying that there was sufficient evidence to suggest an overall relationship between waiting time and the independent variables. Adult models explained more variation in the dependent variable, whereas child models captured less. Given the complex process being modeled, the explanatory power of the models could be considered fair. Models satisfied the assumptions of independence, linearity, homoscedasticity, and normality (Additional File 4 Supporting Information S4).

**TABLE 5:** Linear regression models of waiting time for neurodevelopmental diagnosis for adults.

Covariates	Model A (*n* = 174)		Model B (*n* = 174)	
	Β [95% CI]	*p*	Β [95% CI]	*p*
Quality standards adherence overall score	0.95 [0.31, 2.90]	0.928		
Referral quality standards moderate adherence (vs lower adherence)			0.91 [0.65, 1.28]	0.600
Referral quality standards higher adherence (vs lower adherence)			1.27 [0.60, 1.96]	0.157
Assessment quality standards moderate adherence (vs lower adherence)			2.20 [1.25, 3.90]	0.007*
Assessment quality standards higher adherence (vs lower adherence)			1.08 [0.60, 1.96]	0.800
Demographic factors (any vs. none)	0.85 [0.64, 1.13]	0.257	0.86 [0.67, 1.12]	0.274
Diagnosis autism (vs ADHD)	0.56 [0.39, 0.81]	0.002*	0.69 [0.48, 0.97]	0.034*
Diagnosis other (vs ADHD)	0.78 [0.50, 1.21]	0.269	0.80 [0.53, 1.22]	0.305
History factors lower (vs none)	1.00 [0.74, 1.36]	0.983	1.20 [0.90, 1.60]	0.207
History factors higher (vs none)	0.49 [0.28, 0.88]	0.016*	0.67 [0.39, 1.16]	0.152
No. of contacts 3–4 (vs 1–2)	1.10 [0.77, 1.56]	0.598	1.16 [0.82, 1.62]	0.398
No. of contacts > = 5 (vs 1–2)	0.58 [0.37, 0.90]	0.015*	0.74 [0.49, 1.13]	0.165
	Multiple *R* ^2^ = 0.23 Adjusted *R* ^2^ = 0.19 *p* < 0.001*** *F* = 6.206; *p* < 0.001***		Multiple *R* ^2^ = 0.35 Adjusted *R* ^2^ = 0.31 *p* < 0.001*** *F* = 7.922; *p* < 0.001***	

*Note: N* = 28 cases excluded due to missing or incomplete data. *Quality standards adherence*: Measured across 13 standards (4 pre‐referral, 9 assessment), coded as “Yes” (met) or “No” (not met/unclear). Adherence scores were analyzed as overall (0–13), pre‐referral (0–4), and assessment (0–9). Groups were made by the proportion of standards met for referral and assessment adherence. *Referral adherence levels*: “Lower” (*N* = 76, 0–1 standards met), “Moderate” (*N* = 63, 2 standards met), and “Higher” (*N* = 63, 3–4 standards met). *Assessment adherence levels*: “Lower” (*N* = 25, 0–4 standards met), “Moderate” (*N* = 87, 5–7 standards met), and “Higher” (*N* = 90, 8–9 standards met). *Diagnosis*: ADHD (*N* = 82), Autism (*N* = 70), Other (*N* = 41). *Demographic factors*: “Any” (*N* = 105) includes female sex, minority ethnic background, or a non‐English primary home language; “None” (*N* = 97) indicates none of these were present. *History factors*: Up to eight possible factors, grouped by proportion present: “Lower” (*N* = 83, 1–3 factors present), “Higher” (*N* = 17, 4 or more factors present), and “None” (*N* = 93). *Number of contacts*: Refers to formal diagnostic appointments (face to face, phone, or online): 1–2 (*N* = 57), 3–4 (*N* = 92), > 5 (*N* = 53). *B‐values*: Indicate the factor by which the waiting time changes with the explanatory variable (< 1 denotes a decrease, > 1 an increase, = 1 no change), with *p*‐values indicating statistical significance.

Abbreviations: 95% CI, 95% confidence interval; ADHD, Attention Deficit Hyperactivity Disorder; SD, standard deviation.

* *p* < 0.05.

*** *p* < 0.001.

**TABLE 6:** Linear regression models of waiting time for neurodevelopmental diagnosis for children and adolescents.

Covariates	Model A (*n* = 201)		Model B (*n* = 201)	
	Β [95% CI]	*p*	Β [95% CI]	*p*
Quality standards adherence overall score	0.27 [0.12, 0.62]	0.002**		
Referral quality standards moderate adherence (vs lower adherence)			0.80 [0.60, 1.07]	0.127
Referral quality standards higher adherence (vs lower adherence)			0.70 [0.54, 0.91]	0.008**
Assessment quality standards moderate adherence (vs lower adherence)			1.00 [0.68, 1.49]	0.981
Assessment quality standards higher adherence (vs lower adherence)			0.89 [0.60, 1.33]	0.581
Demographic factors (any vs. none)	0.83 [0.67, 1.03]	0.094	0.85 [0.68, 1.07]	0.160
Diagnosis autism (vs ADHD)	0.78 [0.55, 1.10]	0.150	0.79 [0.55, 1.12]	0.181
Diagnosis autism & ADHD (vs ADHD)	0.89 [0.55, 1.44]	0.630	0.88 [0.54, 1.43]	0.594
Diagnosis other (vs ADHD)	1.09 [0.72, 1.64]	0.694	1.15 [0.75, 1.78]	0.518
History factors lower (vs none)	1.31 [0.99, 1.75]	0.060	1.31 [0.98, 1.75]	0.069
History factors higher (vs none)	1.94 [1.34, 2.80]	< 0.001***	1.99 [1.36, 2.90]	< 0.001***
No. of contacts 3–4 (vs 1–2)	1.02 [0.74, 1.39]	0.919	0.97 [0.70, 1.35]	0.854
No. of contacts > = 5 (vs 1–2)	1.19 [0.88, 1.60]	0.260	1.05 [0.77, 1.45]	0.744
	Multiple *R* ^2^ = 0.13 Adjusted *R* ^2^ = 0.09 *p* < 0.001*** *F* = 3.255; *p* = 0.001***		Multiple *R* ^2^ = 0.12 Adjusted *R* ^2^ = 0.07 *p* = 0.013** *F* = 2.201; *p* = 0.013***	

*Note: N* = 1 case excluded due to missing or incomplete data. *Quality standards adherence*: Measured across 13 standards (4 pre‐referral, 9 assessment), coded as “Yes” (met) or “No” (not met/unclear). Adherence scores were analyzed as overall (0–13), pre‐referral (0–4), and assessment (0–9). Groups were made by the proportion of standards met for referral and assessment adherence. *Referral adherence levels*: “Lower” (*N* = 74, 0–1 standards met), “Moderate” (*N* = 50, 2 standards met), and “Higher” (*N* = 82, 3–4 standards met). *Assessment adherence levels*: “Lower” (*N* = 25, 0–5 standards met), “Moderate” (*N* = 50, 6–7 standards met), and “Higher” (*N* = 82, 8–9 standards met). *Diagnosis*: ADHD (*N* = 25), Autism and ADHD (*N* = 18), Autism (*N* = 126), Other (*N* = 37). *Demographic factors*: “Any” (*N* = 86) includes female sex, minority ethnic background, or a non‐English primary home language; “None” (*N* = 120) indicates none of these were present. *History factors*: Up to seven possible factors, grouped by proportion present: “Lower” (*N* = 130, 1–3 factors present), “Higher” (*N* = 34, 4, or more factors present), and “None” (*N* = 41). *Number of contacts*: Refers to formal diagnostic appointments (face‐to‐face, phone, or online): 1–2 (*N* = 41), 3–4 (*N* = 65), > 5 (*N* = 100). *B‐values*: Indicate the factor by which the waiting time changes with the explanatory variable (< 1 denotes a decrease, > 1 an increase, = 1 no change), with *p*‐values indicating statistical significance.

Abbreviations: 95% CI, 95% confidence interval; ADHD, Attention Deficit Hyperactivity Disorder; SD, standard deviation.

** *p* < 0.01.

*** *p* < 0.001.

For children and adolescents, in multiple model A, treating quality standards as one variable, more adherence to quality standards (overall) was associated with shorter waiting times and an increased number of history factors was associated with longer waiting times. In multiple model B, treating quality standards separately as referral and assessment stages, the highest level of adherence to referral quality standards was associated with shorter waiting times and an increased number of history factors was associated with longer waiting times. For adults, in multiple model A, treating quality standards as one variable, a higher number of history factors, a diagnosis of autism (compared to ADHD), and five or more contacts were associated with reduced waiting times. In multiple model B, treating quality standards as separate referral and assessment stages, a diagnosis of autism (compared to ADHD) was associated with decreased waiting times and a moderate level of adherence to assessment pathway quality standards was associated with increased waiting times.

## 4 Discussion

This study investigated waiting times. Its main advantage is the use of data from a wide range of teams, providing a reasonably accurate snapshot of the current practice across a national population. Good practices were well integrated in child services, with high adherence to standards, compared to slightly lower adherence in adult services. Substantial waits were found in all areas, with children/adolescents experiencing a median wait of 525 days and adults 252 days. Both groups had similar wait times from referral to the first appointment, with around 40% meeting the time standard, although this wait period was longest for adults. Once assessments began, adult services showed greater efficiency, with 73% of cases meeting the 154‐day standard compared to 39% for children/adolescents. Overall, 20% of child cases and 47% of adult cases met the full 252‐day target. More male children/adolescents were included overall, while half of adults were female.

Our findings are in keeping with the broader literature. In Australia, parents (Bent et al. 2020) reported an average waiting period of 12 months and eight appointments to receive an autism diagnosis. Barriers to timely diagnosis included professional advice to “wait and see” delaying formal assessment. Similarly, Crane et al. (2016) found that UK families waited approximately 12 months (365 days) to voice initial concerns about autism and then experienced an average wait of 3.5 years (1280 days) for diagnosis. Over half of these parents expressed dissatisfaction with this and with post‐diagnostic support. The average age for ADHD diagnosis in children ranges between 5 and 9 years (Efron et al. 2013; Sainsbury et al. 2023). Across the UK, France, Germany, Italy, the Netherlands and Spain, the average wait time for ADHD diagnosis is 20.4 months (621 days) (Caci et al. 2014), with the UK having the longest at 31.8 months (967 days) and Spain the shortest at 12.2 months (371 days) (Caci et al. 2014).

In our research, a diagnosis of autism was associated with shorter waits for adults, while an ADHD diagnosis was associated with longer waits (63.4 weeks vs. 38.6 weeks). This shows the more developed local infrastructure for autism, in contrast to the less established processes for ADHD (Asherson et al. 2022; Maciver et al. 2022; Young et al. 2021). The number of history factors, including, for example, the presence of intellectual disability and relevant family history, also emerged as a significant factor. Adults with more complex histories were diagnosed more quickly (32.7 weeks vs. 57.4 weeks), likely due to a perceived higher likelihood of neurodevelopmental differences, leading to speedier triage. In contrast, children/adolescents with increased history complexity experienced longer waits (100.3 weeks vs. 67.7 weeks), possibly due to the need to assess multiple domains and coordinate with families and education settings.

We hypothesized that the demographic factors of female sex, minority ethnicity, and non‐English home language might influence processes, and a combined “risk” category was created. Analysis revealed no significant association with waiting times. However, the lack of diversity in our sample, with ethnic minorities representing only 3.5% of adults and 4.4% of children, limits the analysis. While these figures reflect the ethnic diversity of the Scottish population, the majority of the variance in this variable came from female sex. As noted, we explored using female sex as a standalone variable, but the results were consistent with those obtained using the combined variable. Nevertheless, the data did reveal an underrepresentation of girls in the diagnostic system, with males making up 62% of the assessed population in children's services, and especially in the under‐10 age group where the gender imbalance was more pronounced. This suggests that younger neurodivergent girls are not being identified as well as boys. In contrast, adult services showed a more balanced gender ratio, indicating good recognition in women. These findings raise questions about whether girls need to display more needs or male‐biased diagnostic markers (de Giambattista, Ventura, Trerotoli, Margari, and Margari, 2021) and the continuing need to facilitate good access to diagnosis for adult neurodivergent women (Zener 2019).

Our study investigated the correlation between adherence to quality standards and waiting times, employing models that controlled for demographics, medical history, and diagnosis. A tentative interpretation is that adherence to quality procedures does not inherently lead to delays and instead may be beneficial. In adults, overall adherence was not associated with waiting times, but moderate adherence to assessment standards was associated with longer waits in one model, while the highest adherence levels showed no association. This suggests that there may be a threshold above (or below) where effects are realized. In children/adolescents, higher adherence to overall quality standards and the highest level of adherence at the referral stage standards were associated with shorter waiting times. Moderate adherence level at the referral stage was not related to waiting times, again indicating a potential threshold where greater adherence may be necessary to yield an impact. Findings suggest for children's services that adhering to standards around robust initial pre‐referral practice could streamline pathways and reduce waiting times by ensuring comprehensive early data. For adults, interpreting the situation is more complex, particularly due to using child‐focused standards. While levels of adherence to standards do not appear to negatively impact waiting times, adult assessments face challenges, including a limited evidence base for diagnostic tools and difficulty identifying family and community informants. These issues highlight the need for adult‐specific quality standards.

Two further issues warrant attention. First, the low identification of Fetal Alcohol Spectrum Disorder (FASD). Despite its prevalence, thought to be at least as common as autism (Mukherjee 2021; Popova et al. 2023), FASD was only diagnosed once in our sample (Chamberlain et al. 2017; Temple et al. 2021). A second important factor is the impact of the COVID‐19 pandemic. The pandemic heightened the demand for neurodevelopmental assessments. During lockdowns, increased stress and proximity led many individuals to recognize neurodivergence in themselves and others. While adult services could transition to online assessment, such adaptations were less feasible for children. Some children's services ceased all neurodevelopmental assessments during the pandemic. These factors coincided with a significantly heightened societal awareness of neurodiversity, leading to an exceptionally high demand for services in 2021/2022 and influencing some of the high waiting times seen in this study.

## 5 Recommendations

Our research shows that diagnostic processes take significant time, with service quality factors being at least as influential as individual‐level factors. As service factors are within our control, we should focus on addressing them. Key measures to improve waiting experiences might include a single point of access, standardized referral formats, or helplines/online resources for immediate support. Clear time standards, with a commitment to adherence, may also improve consistency and accountability. For children, school‐based assessments and teacher training strengthen the care network, while adult services benefit from engaging GPs and mental health practitioners. Given the long waits observed, comprehensive, integrated pathways addressing the full spectrum of neurodevelopmental differences could offer another solution (Fleming et al. 2020; Male, Farr, Allard, et al. 2023; Rivard et al. 2021; Rodgaard et al. 2021). Many autistic children show additional neurodivergent traits, but few undergo further evaluation (Lang et al. 2024). As neurodevelopmental differences very often co‐occur, current pathways focusing on single conditions may be leading to inefficiencies, delays, duplication, and longer waits (Male, Farr, Allard, et al. 2023; Male, Farr, and Reddy, 2020). Recent Scottish data show that 16.8% of school children are neurodivergent (Maciver et al. 2023), revealing a gap in current models that focus on lower expected prevalence for autism and ADHD. Our research found that 83%–87% of individuals assessed received a neurodevelopmental diagnosis, although most were given only one diagnosis. This is similar to other UK studies (Lang et al. 2024; Male, Farr, Bremner, et al. 2023; Parr et al. 2021). Adult services in the UK mainly address autism and ADHD separately, often only for individuals in crisis. A transdiagnostic approach that removes rigid criteria for visible distress would eliminate the need for multiple specialist consultations and repeated assessments, enabling earlier support and reducing the need for crisis interventions. Such pathways are emerging in Scotland and advocated for in a recent UK‐wide study focusing on autism (Abrahamson et al. 2021), with similar approaches discussed in the US, Canada, and Australia (Penner et al. 2023; Rivard et al. 2021; Whitehouse et al. 2018; Zwaigenbaum et al. 2021). Services moving toward using transdiagnostic pathways will need to address costs, workforce planning, and service integration. Training programs, local strategy groups that include neurodivergent individuals, increased AHPs in assessment teams, and dedicated neurodevelopmental assessment units might also form part of the solution.

Future efforts should prioritize improving the assessment experience by actively listening to neurodivergent individuals. This particularly requires developing a clear understanding of what defines a neuro‐affirming assessment, diagnostic pathway, and overall experience. Neurodevelopmental differences are lifelong but need not be viewed through a deficit lens. Limiting diagnoses to those who appear severely affected is ethically wrong and denies individual support, peers, and positive self‐identity (Arnold et al. 2020; de Broize et al. 2022; Lilley et al. 2022; McGill et al. 2020; Zener 2019). Diagnosis should therefore be available to all who need it, irrespective of visible impact, to provide help and reduce escalation of need. One final solution involves reviewing different, potentially more radical models, including pathways that incorporate pragmatic, consensus‐based, brief, or self‐identification, with extended assessments and greater professional involvement available when necessary (Fletcher‐Watson 2024). Here, diagnostic formulations should focus not only on identifying labels but also on understanding strengths and everyday support needs.

## 6 Limitations

Reliance on voluntary participation and quota sampling may not have captured a representative cross section of the population and lacked randomization. While this methodology minimized technical burdens on staff, it is possible that teams under severe operational strain, which might have longer wait times, opted out. Given the clinical time required for data extraction, we carefully selected variables, though this constrained the models and may have left confounding factors unaccounted for. Relevant variables may include age, parental education, and income. Age was recorded but excluded from primary analyses to ensure model comparability. While important for children, age was considered less significant for adults. Also, multiple age measures exist (referral, assessment, diagnosis), but assessment and diagnosis age are direct functions of waiting time, which could introduce circular reasoning. Younger children may receive quicker diagnoses due to more pronounced needs, but this was already captured by our history factor variable. Parental education and income were unavailable in clinical notes. In the modeling, where no pre‐existing rationale or established cut points were available, the data were categorized to create balanced groups rather than relying on previously derived thresholds. We also considered subdividing the “children” category to examine age‐related differences in waiting times but opted for broader age groups to preserve sample size. Additionally, the services are the same; there are some specialist aspects of service for very young children but most services take children from birth to 18 years. The comparison of waiting times between children and adult services also presents challenges. Children's pathways tend to be more inclusive, while adult pathways are more restrictive, often excluding individuals who do not have moderate to severe mental illnesses. This means that adult services are generally focused on a smaller, more specific subset of individuals. Another limitation is the use of pediatric standards for adult services. Additionally, some regions may have refrained from participation due to the lack of established adult autism or ADHD pathways. These disparities between children's and adult services make it difficult to draw direct comparisons, and the observed higher efficiency in adult services should be interpreted with caution. Positive outcomes for adults may not fully represent the broader context, where significant access challenges remain. In some cases, services dismiss referrals rather than placing them on waiting lists, a provision gap that we did not investigate.

## 7 Conclusion

Long waiting times were observed. There were some efficiencies, with 73% of adult and 39% of child cases meeting the 154‐day assessment time standard. Demographic factors were not associated with waiting times, but children/adolescents with complex medical or developmental backgrounds experienced longer waits, while adults with similar profiles progressed more quickly. Adherence to quality standards did not appear to increase waits and could instead have a beneficial effect. Tailored standards for adult services are needed. Most individuals received a single diagnosis, with fewer cases of co‐occurring diagnoses. Gender representation was balanced among adults, but more boys than girls were referred for assessment in children's services.

## Ethics Statement

Ethics approval for the study was obtained from the Queen Margaret University Ethics Committee and the NHS Scotland Public Benefit and Privacy Panel for Health and Social Care.

## Conflicts of Interest

The authors declare no conflicts of interest.

## Supporting information


Data S1.

## Data Availability

The data that support the findings of this study are available on request from the corresponding author. The data are not publicly available due to privacy or ethical restrictions.
