# Supplementary material for: Waiting Times and Influencing Factors in Children and Adults Undergoing Assessment for Autism, ADHD, and Other Neurodevelopmental Differences
Source: Autism Res. 2025 Feb 24;18(4):788–801. doi: 10.1002/aur.70011 (PMC12015800; doi:10.1002/aur.70011)

Contents

[Additional file 1: List of quality standards and levels of adherence for child and adult services 2](#_Toc183611057)

[Additional file 2: Variables used for modelling with distributions of the variables as used in the analysis 4](#_Toc183611058)

[Additional file 3: More info on diagnosis 11](#_Toc183611059)

[Additional file 4: Model Assumption Checks 12](#_Toc183611060)

Tables

[Table 1: Quality Standards 2](#_Toc184632192)

[Table 2: Time standards 3](#_Toc184632193)

[Table 3: Diagnosis variables for analysis CHILD 5](#_Toc184632194)

[Table 4: Demographic factor variables for analysis CHILD 5](#_Toc184632195)

[Table 5: History factor variable intervals and categories for analysis CHILD 5](#_Toc184632196)

[Table 6: Referral pathway variable intervals and categories for analysis CHILD 6](#_Toc184632197)

[Table 7: Assessment pathway variable intervals and categories for analysis CHILD 6](#_Toc184632198)

[Table 8: Number of contacts variable intervals and categories for analysis CHILD 6](#_Toc184632199)

[Table 9: Overall quality adherence variable (score used for analysis) CHILD 7](#_Toc184632200)

[Table 10: Diagnosis variables for analysis ADULT 8](#_Toc184632201)

[Table 11: Demographic factor variable categories for analysis ADULT 8](#_Toc184632202)

[Table 12: History factor variable intervals and categories for analysis adult 8](#_Toc184632203)

[Table 13: Referral pathway intervals and categories for analysis ADULT 9](#_Toc184632204)

[Table 14: Assessment pathway variable intervals and categories for analysis ADULT 9](#_Toc184632205)

[Table 15: Number of contacts variable intervals and categories for analysis ADULT 9](#_Toc184632206)

[Table 16: Overall quality adherence variable (score used for analysis) ADULT 10](#_Toc184632207)

# Additional file 1: List of quality standards and levels of adherence for child and adult services

Table 1: Quality Standards

| **Stage in Pathway** | **Quality process standard** | **Child (n=206)**  **N times standard met (%)** | | **Adult (n=202)**  **N times standard met (%)** | |
| --- | --- | --- | --- | --- | --- |
| Pre-referral standards | The pre-referral process included a developmental and family history | 158 | (76.70) | 121 | (59.90) |
|  | The pre-referral process included information drawn from a variety of contexts, such as home, school, workplace and the community | 190 | (92.23) | 94 | (46.53) |
|  | The pre-referral process included indicators for concern identified through a screening tool | 108 | (52.43) | 143 | (70.79) |
|  | The pre-referral process included information drawn from direct observation or interview with the individual | 113 | (54.85) | 104 | (51.49) |
| Assessment standards | The neurodevelopmental assessment was conducted by more than one person as part of an MDT | 163 | (79.13) | 84 | (41.58) |
|  | Diagnosis was be made with reference to recognized diagnostic criteria (DSM or ICD) | 157 | (76.21) | 142 | (70.30) |
|  | At time of diagnosis, post diagnosis information was given | 182 | (88.35) | 150 | (74.26) |
|  | A post-diagnosis follow-up meeting was offered | 70 | (33.98) | 161 | (79.70) |
|  | The neurodevelopmental diagnostic assessment included history taking | 184 | (89.32) | 183 | (90.59) |
|  | The neurodevelopmental diagnostic assessment used information drawn from observation of the individual | 187 | (90.78) | 147 | (72.77) |
|  | The neurodevelopmental diagnostic assessment included a parent/carer/partner/family member | 160 | (77.67) | 82 | (40.59) |
|  | The neurodevelopmental diagnostic assessment used information drawn from a variety of contexts, such as home, school, workplace and community | 188 | (91.26) | 150 | (74.26) |
|  | The neurodevelopmental diagnostic assessment used information drawn from standardised or other formal tool | 167 | (81.07) | 139 | (68.81) |

Table 2: Time standards

| **Stage in Pathway** | **Time Standard** |
| --- | --- |
| *Request for neurodevelopmental assessment (pre referral)* | Time from acceptance of referral to first appointment should be no longer than: **12 weeks (84 days)** |
| *Diagnostic assessment* | Time from first appointment to last appointment should be no longer than: **22 weeks (154 days)** |
| *Full process* | Total waiting time (from request/referral accepted to diagnosis shared) should be no longer than: **36 weeks (252 days)** |

# Additional file 2: Variables used for modelling with distributions of the variables as used in the analysis

**Data for children**

Table 3: Diagnosis variables for analysis CHILD

| **Category** | **N** |
| --- | --- |
| ADHD with or without another diagnosis (excluding Autism) | 25 |
| Autism and ADHD combined, with or without another diagnosis | 18 |
| Autism with or without another diagnosis (excluding ADHD) | 126 |
| Other | 37 |

Table 4: Demographic factor variables for analysis CHILD

| **Category** | **N** |
| --- | --- |
| None | 120 |
| Any | 86 |

Table 5: History factor variable intervals and categories for analysis CHILD

| **Category** | **Interval** | **N** |
| --- | --- | --- |
| None: N=41 | 0.00 | 41 |
| Lower: N=130 | 0.13 | 27 |
|  | 0.14 | 11 |
|  | 0.17 | 1 |
|  | 0.20 | 2 |
|  | 0.25 | 22 |
|  | 0.29 | 13 |
|  | 0.33 | 7 |
|  | 0.38 | 24 |
|  | 0.40 | 2 |
|  | 0.43 | 21 |
| Higher: N=34 | 0.50 | 17 |
|  | 0.57 | 4 |
|  | 0.60 | 1 |
|  | 0.63 | 3 |
|  | 0.67 | 3 |
|  | 0.71 | 3 |
|  | 0.80 | 1 |
|  | 0.86 | 1 |
|  | 1.00 | 1 |

N=1 missing data

Table 6: Referral pathway variable intervals and categories for analysis CHILD

| **Category** | **Interval** | **N** |
| --- | --- | --- |
| Lower: N=74 | 0 | 28 |
|  | 0.25 | 46 |
| Moderate: N=50 | 0.5 | 50 |
| Higher: N=82 | 0.75 | 46 |
|  | 1 | 36 |

Table 7: Assessment pathway variable intervals and categories for analysis CHILD

| **Category** | **Interval** | **N** |
| --- | --- | --- |
| Lower: N=25 | 0.11 | 1 |
|  | 0.22 | 3 |
|  | 0.33 | 5 |
|  | 0.44 | 5 |
|  | 0.56 | 11 |
| Moderate: N=57 | 0.67 | 26 |
|  | 0.78 | 31 |
| Higher: N=124 | 0.89 | 86 |
|  | 1.00 | 38 |

Table 8: Number of contacts variable intervals and categories for analysis CHILD

| **Category** | **Interval** | **N** |
| --- | --- | --- |
| 1-2: N=41 | 1 | 13 |
|  | 2 | 28 |
| 3-4: N=65 | 3 | 36 |
|  | 4 | 29 |
| >5: N=100 | 5 | 34 |
|  | 6 | 30 |
|  | 7 | 14 |
|  | 8 | 9 |
|  | 9 | 4 |
|  | 10 | 9 |

Table 9: Overall quality adherence variable (score used for analysis) CHILD

| **Score** | **N** |
| --- | --- |
| 0.15 | 1 |
| 0.31 | 3 |
| 0.38 | 3 |
| 0.46 | 6 |
| 0.54 | 13 |
| 0.62 | 33 |
| 0.69 | 38 |
| 0.77 | 57 |
| 0.85 | 31 |
| 0.92 | 18 |
| 1.00 | 3 |

Data for adults

Table 10: Diagnosis variables for analysis ADULT

| **Category** | **N** |
| --- | --- |
| ADHD with or without another diagnosis (excluding Autism) | 82 |
| Autism and ADHD combined, with or without another diagnosis | 9 |
| Autism with or without another diagnosis (excluding ADHD) | 70 |
| Other | 41 |

Table 11: Demographic factor variable categories for analysis ADULT

| **Category** | **N** |
| --- | --- |
| None | 97 |
| Any | 105 |

Table 12: History factor variable intervals and categories for analysis ADULT

| **Category** | **Interval** | **N** |
| --- | --- | --- |
| None: N=93 | 0.00 | 93 |
| Lower: N=83 | 0.13 | 26 |
|  | 0.14 | 13 |
|  | 0.17 | 3 |
|  | 0.20 | 4 |
|  | 0.25 | 9 |
|  | 0.29 | 4 |
|  | 0.33 | 4 |
|  | 0.38 | 18 |
|  | 0.40 | 1 |
|  | 0.43 | 1 |
| Higher: N=17 | 0.50 | 8 |
|  | 0.60 | 1 |
|  | 0.63 | 2 |
|  | 0.67 | 1 |
|  | 0.75 | 4 |
|  | 0.88 | 1 |

N=9 missing data

Table 13: Referral pathway intervals and categories for analysis ADULT

| **Category** | **Interval** | **N** |
| --- | --- | --- |
| Lower: N=76 | 0 | 27 |
|  | 0.25 | 49 |
| Moderate: N=63 | 0.5 | 63 |
| Higher: N=63 | 0.75 | 30 |
|  | 1 | 33 |

Table 14: Assessment pathway variable intervals and categories for analysis ADULT

| **Category** | **Interval** | **N** |
| --- | --- | --- |
| Lower: N=25 | 0.11 | 1 |
|  | 0.22 | 4 |
|  | 0.33 | 6 |
|  | 0.44 | 14 |
| Moderate: N=87 | 0.56 | 26 |
|  | 0.67 | 40 |
|  | 0.78 | 21 |
| Higher: N=90 | 0.89 | 54 |
|  | 1.00 | 36 |

Table 15: Number of contacts variable intervals and categories for analysis ADULT

| **Category** | **Interval** | **N** |
| --- | --- | --- |
| 1-2: N=57 | 1 | 7 |
|  | 2 | 50 |
| 3-4: N=92 | 3 | 64 |
|  | 4 | 28 |
| >5: N=53 | 5 | 18 |
|  | 6 | 28 |
|  | 7 | 4 |
|  | 8 | 1 |
|  | 9 | 1 |
|  | 10 | 1 |

Table 16: Overall quality adherence variable (score used for analysis) ADULT

| **Score** | **N** |
| --- | --- |
| 0.15 | 1 |
| 0.23 | 4 |
| 0.31 | 6 |
| 0.38 | 8 |
| 0.46 | 9 |
| 0.54 | 20 |
| 0.62 | 32 |
| 0.69 | 42 |
| 0.77 | 43 |
| 0.85 | 28 |
| 0.92 | 8 |
| 1.00 | 1 |

# Additional file 3: More info on diagnosis

**Diagnoses received (counts of children, including combinations) following assessment were:**

- ADHD alone n=17 (8.25%)
- Autism alone n=108 (52.43%)
- Developmental Coordination Disorder alone n=1 (0.49%)
- Developmental Language Disorder alone n=2 (0.97%)
- Intellectual Disability alone n=5 (2.43%)
- ADHD & Developmental Coordination Disorder n=3 (1.46%)
- ADHD & Intellectual Disability n=2 (0.97%)
- ADHD & Other n=3 (1.46%)
- Autism & ADHD & Developmental Coordination Disorder n=1 (0.49%)
- Autism & ADHD & Intellectual Disability n=1 (0.49%)
- Autism & ADHD & Other n=2 (0.97%)
- Autism & ADHD n=14 (6.80%)
- Autism & Developmental Language Disorder & ID n=1 (0.49%)
- Autism & Intellectual Disability & Other n=1 (0.49%)
- Autism & Intellectual Disability n=7 (3.40%)
- Autism & Other n=9 (4.37%)
- Other alone n=2 (0.97%)
- No diagnosis on completion of assessment n=28 (13.59%)
  - **65.53% of the children had one diagnosis, 21.36% of the children had multiple diagnoses.**

**Diagnoses received (counts of adults, including combinations) following assessment were**

- Autism alone n=69 (34.16%)
- ADHD alone n=63 (31.19%)
- Intellectual Disability alone n=9 (4.46%)
- ADHD & Other n=19 (9.41%)
- Autism & ADHD n=6 (2.97%)
- Autism & ADHD & Other n=2 (0.99%)
- Autism & Other n=1 (0.50%)
- Autism & ADHD & Developmental Language Disorder & Intellectual Disability & FASD n=1 (0.50%)
- Developmental Language Disorder & Intellectual Disability n=1 (0.50%)
- Other alone n=4 (1.98%)
- No diagnosis on completion of assessment n=28 (13.86%)
  - **71.78% of the adults had one diagnosis, 14.85% of adults had multiple diagnoses**

# Additional file 4: Model Assumption Checks

**Model A (Adult)**


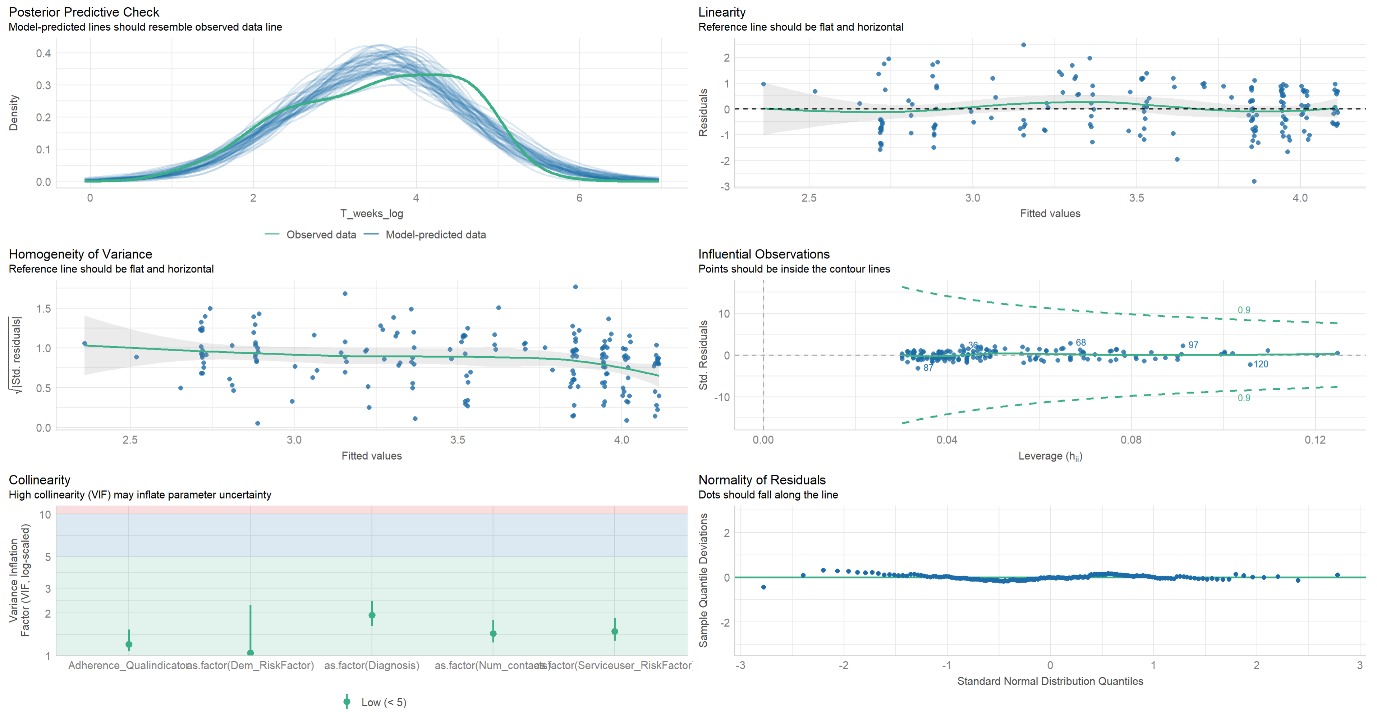


**Model B (Adult)**


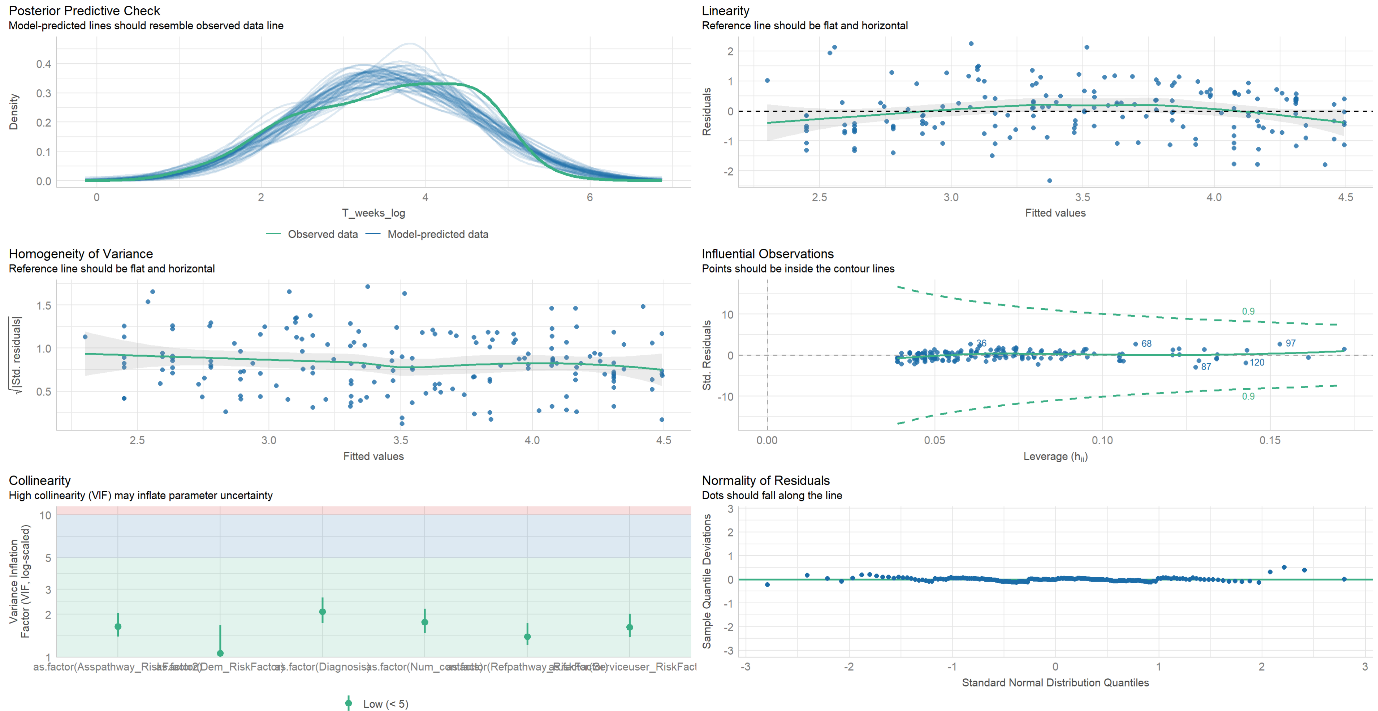


**Model A (Child)**


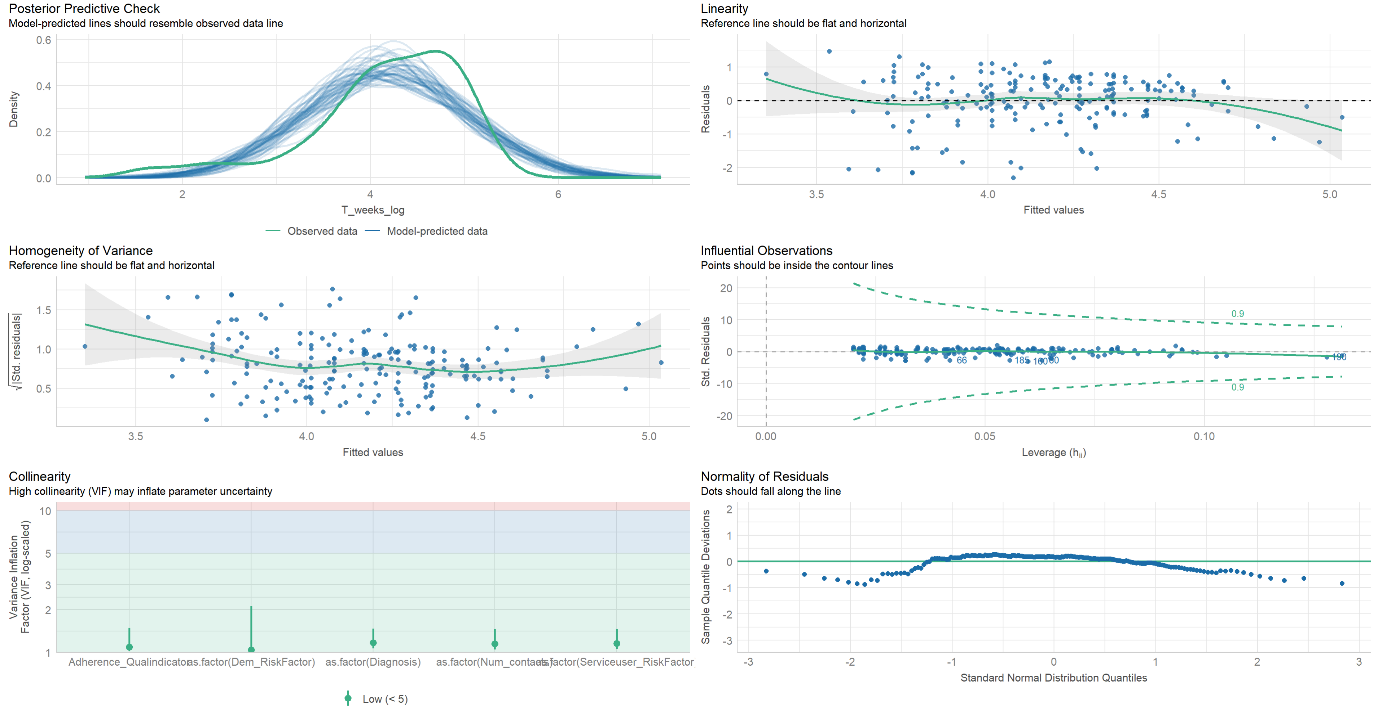


**Model B (Child)**


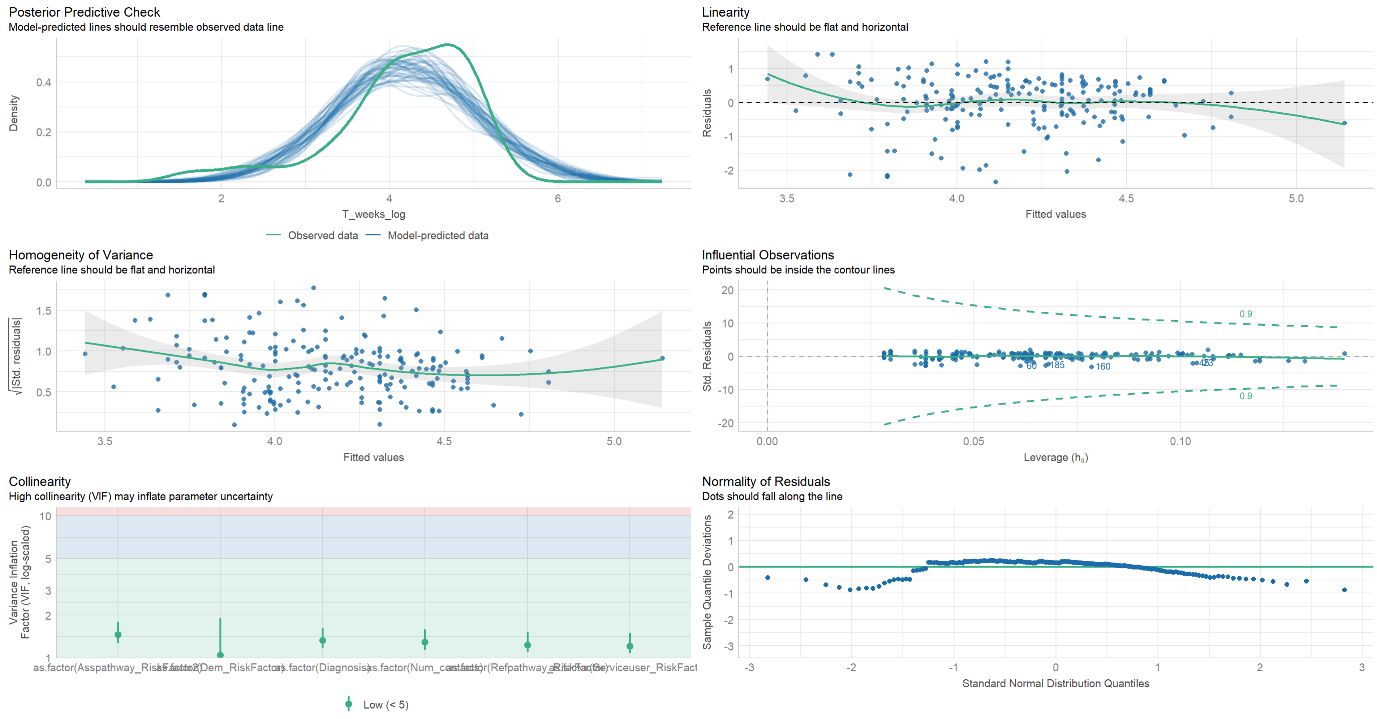

Supplement: Supplementary file 1 — Data S1. [file AUR-18-788-s001.docx]
